# Supplementary material for: Senescence-Independent Anti-Inflammatory Activity of the Senolytic Drugs Dasatinib, Navitoclax, and Venetoclax in Zebrafish Models of Chronic Inflammation
Source: Int J Mol Sci. 2022 Sep 9;23(18):10468. doi: 10.3390/ijms231810468 (PMC9499634; doi:10.3390/ijms231810468)
Supplement: Supplementary file 1 [file ijms-23-10468-s001.zip › ijms-1835580-supplementary.pptx]

## Slide 1
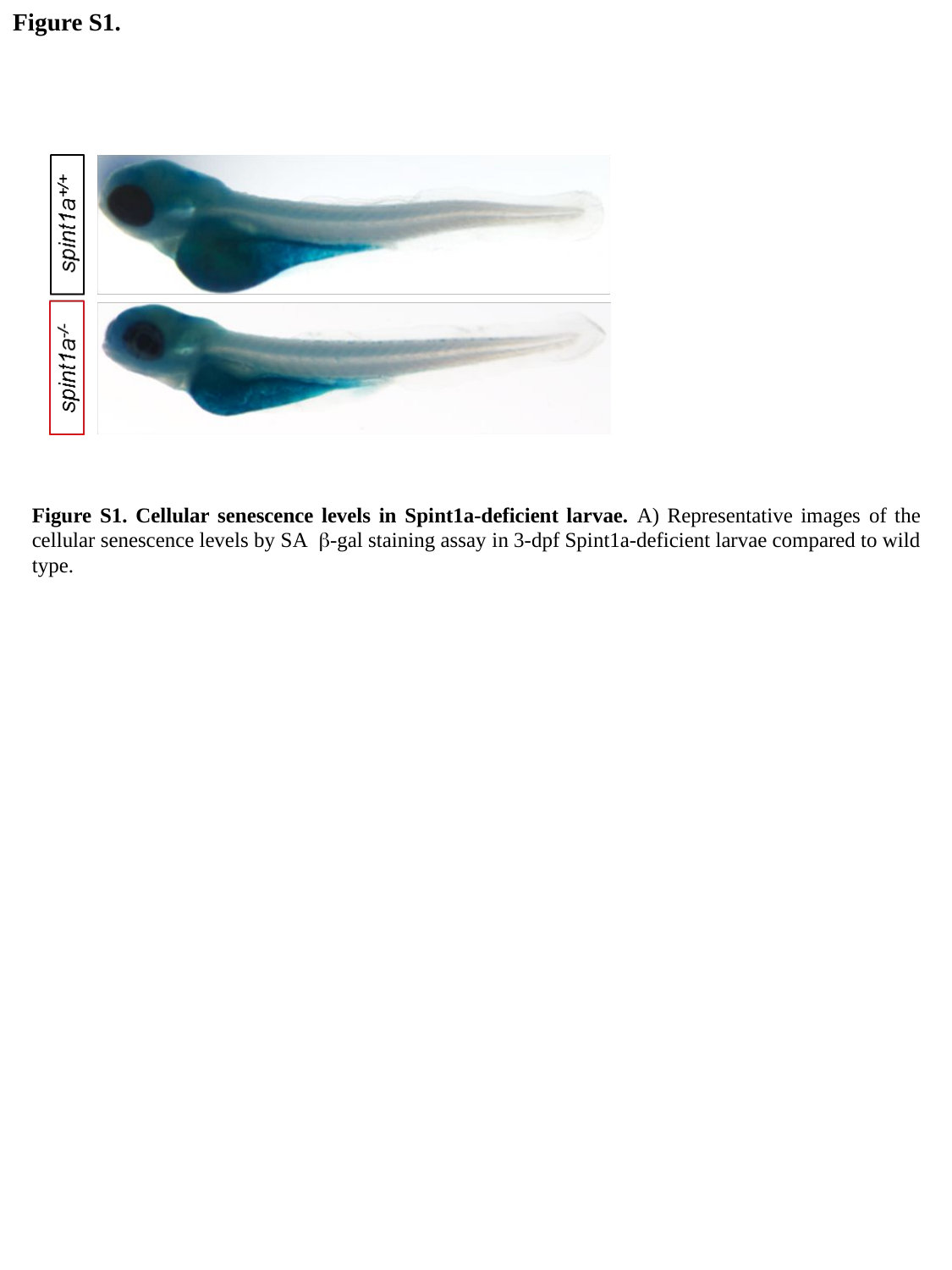

Figure S1.
Figure S1. Cellular senescence levels in Spint1a-deficient larvae. A) Representative images of the cellular senescence levels by SA b-gal staining assay in 3-dpf Spint1a-deficient larvae compared to wild type.

## Slide 2
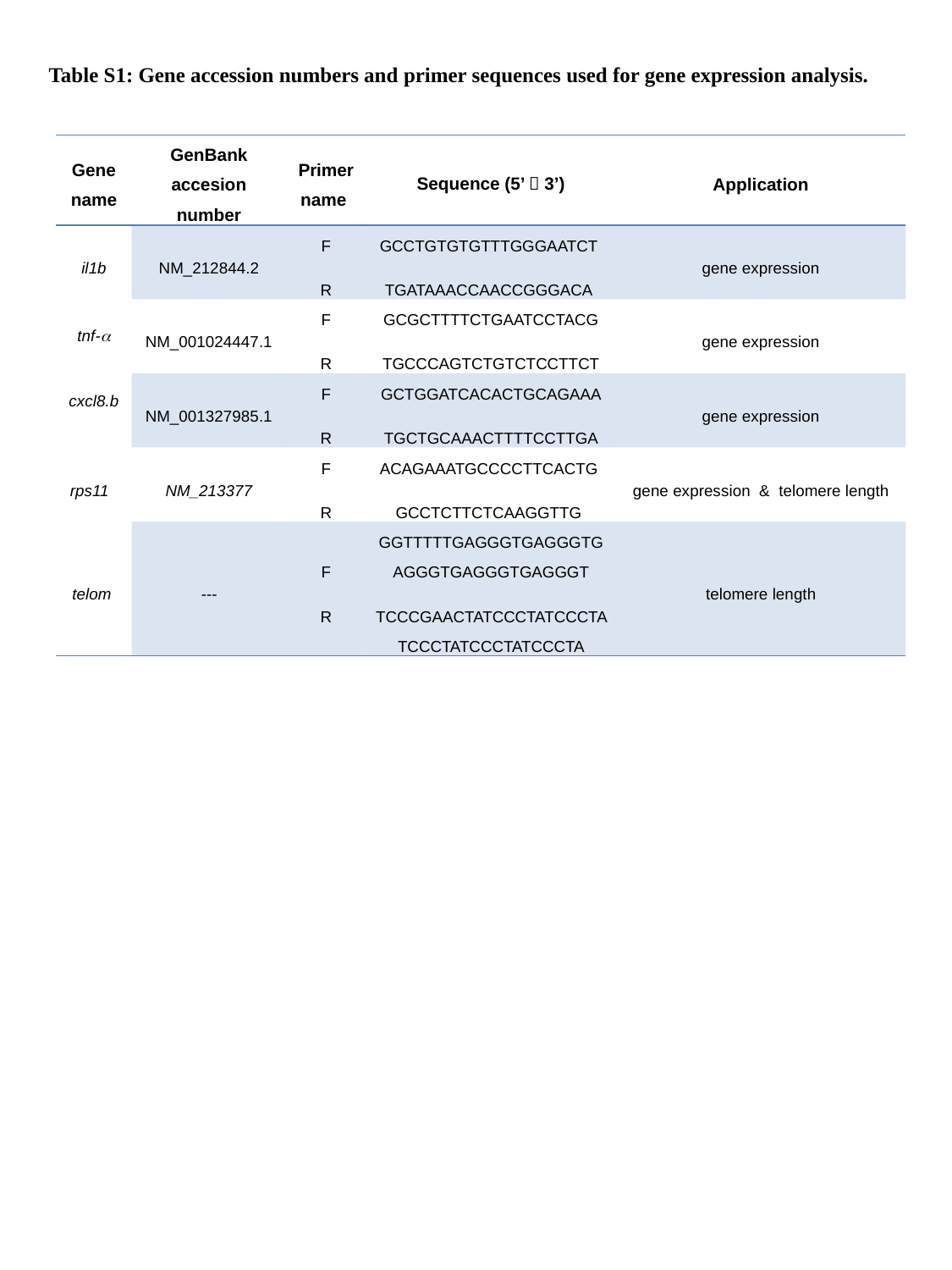

Table S1: Gene accession numbers and primer sequences used for gene expression analysis.
| Gene name | GenBank accesion number | Primer name | Sequence (5’  3’) | Application |
| --- | --- | --- | --- | --- |
| il1b | NM\_212844.2 | F R | GCCTGTGTGTTTGGGAATCT  TGATAAACCAACCGGGACA | gene expression |
| tnf-α | NM\_001024447.1 | F R | GCGCTTTTCTGAATCCTACG TGCCCAGTCTGTCTCCTTCT | gene expression |
| cxcl8.b | NM\_001327985.1 | F R | GCTGGATCACACTGCAGAAA TGCTGCAAACTTTTCCTTGA | gene expression |
| rps11 | NM\_213377 | F R | ACAGAAATGCCCCTTCACTG  GCCTCTTCTCAAGGTTG | gene expression & telomere length |
| telom | --- | F R | GGTTTTTGAGGGTGAGGGTGAGGGTGAGGGTGAGGGT TCCCGAACTATCCCTATCCCTATCCCTATCCCTATCCCTA | telomere length |
